# Examining the Cultural Appropriateness and Acceptability of a Traditional Birth Attendants’ Training Program in Rural Guatemala

**DOI:** 10.7759/cureus.69940

**Published:** 2024-09-22

**Authors:** Yulia Shtanko, Melissa N Litenski, Rachel Clarke, Sasha Hernandez, Jessica B Oliveira

**Affiliations:** 1 Medical Education, Florida International University, Herbert Wertheim College of Medicine, Miami, USA; 2 Psychiatry and Behavioral Health, Florida International University, Herbert Wertheim College of Medicine, Miami, USA; 3 Obstetrics and Gynecology, NYU (New York University) Langone Health, New York City, USA

**Keywords:** maternal and infant mortality, obstetrics, rural guatemala, traditional birthing attendants, underserved populations

## Abstract

Introduction: While there has been notable global advancement in reducing maternal mortality rates (MMRs) in Latin America, the rates among indigenous women remain alarmingly high. This disparity persists in Guatemala, where indigenous women face a two-fold higher MMR compared to their non-indigenous counterparts. Most of the obstetrical care is performed by traditional Mayan birth attendants (TBAs), also known as comadronas, who have minimal formalized clinical training in obstetrical care. Considering there was no national comprehensive training program for TBAs, a unique training program was established in 2014. This program, the School of PowHER (Providing Outreach in Women’s Health and Educational Resources), aims to ensure sustainable education led by TBAs for TBAs in rural Guatemala with the ultimate goal of helping TBAs provide basic antenatal care and learn how to identify and refer high-risk pregnancies. The aim of this proposed study is to examine the cultural appropriateness and sensitivity of the training program through a mixed-methods approach.

Methods: We utilized a mixed-methods strategy, combining quantitative and qualitative methodologies. The quantitative aspect involved a 14-item written survey using a three-point Likert scale for responses, while the qualitative part utilized a semi-structured interview guide to conduct a focus group discussion.

Results: The survey (n=33) showed that 32 comadronas found the curriculum applicable (97%) and comprehensible (97%). However, only 26 comadronas (79%) were comfortable with anatomy terminology. Opinions on teaching tools varied: 13 comadronas (39%) felt they were always representative, 13 comadronas (39%) sometimes, and seven comadronas (21%) never. Group discussions echoed this lack of representation. In the learning environment, 32 comadronas felt welcomed (97%) and 31 felt understood (94%), but five comadronas (15%) were not comfortable asking questions. Thirty-one comadronas (94%) believed training made pregnant women trust comadronas more. Group interviews highlighted increased confidence, better care, and perceived lower maternal mortality.

Conclusion: This study found the program to be culturally sensitive and effective. Group interviews highlighted increased confidence, improved patient care, and perceived reductions in maternal mortality. Feedback emphasized the need for more culturally relevant materials, resources, and collaboration with the Ministry of Health. This program's community-centered approach could serve as a model for similar initiatives in low- and middle-income countries addressing high maternal mortality rates, despite language and access challenges.

## Introduction

Although significant worldwide progress has been made toward lowering maternal mortality rates (MMRs) in Latin America, rates for indigenous women continue to be unacceptably high [1]. This holds true in Guatemala, where indigenous women experience a two-fold higher MMR compared to non-indigenous women. The causes of this disparity are complex and multifaceted; they include generational social and economic exclusion, limited education, disadvantageous gender roles, and lack of mastery of Spanish, among other cultural factors [2]. These structural determinants of health, coupled with the fact that over 80% of Mayan women opt to give birth at home foregoing formal healthcare facilities for prenatal care due to a seated distrust of institutional care, ultimately lead to worse outcomes during pregnancy and childbirth [3].

In Guatemala, improving MMR among Mayan women presents a multifaceted challenge. A promising approach within this complex landscape involves harnessing the expertise of traditional birth attendants (TBAs), known locally as comadronas. Mayan women heavily rely on comadronas throughout their pregnancy and childbirth journey, seeking culturally appropriate care delivered in their preferred Mayan language [4,5]. Comadronas also facilitate traditional birthing practices, including steam baths and herbal remedies, often not accommodated in local health facilities. Given the role comadronas hold with the community, when they are effectively integrated into the existing health system, this cultural acceptance within an established health system can yield positive pregnancy and childbirth outcomes [1,6-8].

In the Lake Atitlan region of Guatemala, where MMR among Mayan women remains high, the imperative to bridge cultural barriers between comadronas and healthcare professionals must be met with innovative solutions [9]. The School of PowHER (Providing Outreach in Women’s Health and Educational Resources), established in 2014, presents a transformative model of comadrona recruitment and training based on WHO guidelines and whose content has been shaped by local and international experts [10]. The School of PowHER approach not only equips comadronas with essential skills in basic prenatal care and safe labor practices but also fosters values of knowledge sharing, apprenticeship, and collaboration with local health facility providers [9]. By directly addressing critical aspects contributing to labor complications in indigenous communities, such as recognizing complications and deciding to seek care, the School of PowHER's curriculum demonstrates a commitment to improving maternal healthcare outcomes by addressing culturally relevant care. In addition, the School of PowHER collaborates with the Ministry of Public Health and Social Welfare of the Republic of Guatemala (MSPAS) to identify comadrona students from areas of need.

To date, the School of PowHER stands as a sustainable model of care led by local nurses and comadronas, demonstrating notable success in enhancing prenatal visits, increasing referrals, and augmenting knowledge of basic prenatal care and safe labor among its trained comadronas [10]. However, despite its evident achievements, the formal evaluation of its cultural sensitivity remains unexplored. As the School of PowHER continues to evolve, maintaining cultural appropriateness emerges as a critical priority, given the profound impact of cultural nuances on the individuals most directly involved, the students themselves. Therefore, this study aims to assess the cultural sensitivity of the School of PowHER model of care in Guatemala's Lake Atitlan region, focusing on evaluating its alignment with local cultural norms and values. By addressing this gap in the literature on educational programs for TBAs, this research seeks to contribute to the ongoing evolution of the School of PowHER program, ensuring its continued effectiveness and relevance in promoting maternal health among Mayan women and helping inform other similar TBA programs in low-middle-income countries.

## Materials and methods

Study design

This observational study employed a mixed-methods approach, integrating both quantitative and qualitative methodologies via a convergent parallel mixed-methods study design. The quantitative component utilized a 14-question written survey incorporating a three-point Likert scale for responses, while the qualitative arm employed a semi-structured interview guide to facilitate a focus group discussion. The survey was created by the research team in close collaboration with school staff, who offered valuable input on key questions and areas for evaluation. After finalization, it was translated into Spanish by a native speaker, then verified through back-translation by another native speaker and further validated with computerized translation tools.

Participants

The study involved graduates from the School of PowHER, encompassing eight graduating classes with a total of 102 trained comadronas. Eligibility criteria for participation included (a) continued engagement in patient care since graduation and (b) ability to attend the primary research site, the town of Santiago on Lake Atitlan, or participate in a phone interview. Out of the 102 graduates, 33 met the specified criteria. Reasons for non-participation varied, ranging from challenges in contacting comadronas to transportation barriers and prior family responsibilities. Selected participants were proficient in moderate to fluent Spanish, aged 55 years or younger, licensed by the Guatemalan Ministry of Health and Public Assistance (MSPAS), and capable of committing time to the program.

Data collection

Over the span of a week in the month of March, year 2023, eligible participants were invited to the research site, the School of PowHER campus in the city of Santiago Atitlan, to complete the 14-question survey assessing the program's cultural sensitivity and perceived impact. Following completion of the survey, focus group discussions were conducted guided by open-ended questions to explore the alignment of the curriculum with cultural backgrounds, participants' preparedness to apply acquired skills, and areas for improvement. The data was collected by filling out a physical questionnaire for the 14-question survey. The researchers recorded, transcribed, and analyzed the focus group discussions.

Analysis

Quantitative data from the 14-question survey underwent descriptive analyses, calculating average percentages of responses. Qualitative data from the focus group discussions were also analyzed, generating insights into participants' perspectives. This was done by transcribing the focus group discussion and reading the transcriptions and mapping for themes regarding cultural appropriateness, comfort with the program, and suggested improvements. Results from both the quantitative and qualitative analyses were assessed to provide a comprehensive understanding of the program's cultural sensitivity and impact.

## Results

Questionnaire results

Table 1 presents demographic information for the 33 participants included in the study. The average age of participants is 46 years, with a standard deviation of 16 years, while their average years of experience is 15, with a standard deviation of 12 years. The majority of participants (58%) are comadronas with other roles, including comadronas who also serve as educators (12%), professional nurses (6%), and various other roles. Participants speak various languages, with Tzʼutujil and Spanish being the most common (49%). Most participants (70%) are literate, with varying levels of education, ranging from none to university degrees. This demographic overview provides insight into the diverse backgrounds of participants involved in the study.

**Table 1:** Participant Demographics (N=33 participants) The participant demographics indicate their age, years of experience, current employment, languages spoken, literacy, and education level. * For Saving Mothers.

Characteristic	No. (%)
Age	46±16
Years of experience	15±12
Current employment	
Comadrona*	19 (58%)
Comadrona* and educator	4 (12%)
Professional nurse	2 (6%)
Comadrona* and auxiliary nurse	2 (6%)
Auxiliary nurse	4 (12%)
Educator* and administrator*	1 (3%)
Health facilitator	1 (3%)
Languages spoken	
Tzʼutujil and Spanish	16 (49%)
Spanish and Quiche	5 (15%)
Tzʼutujil	4 (12%)
Spanish	2 (6%)
Spanish and Kakchiquel	2 (6%)
Tzʼutujil, Spanish, and Quiche	2 (6%)
Tzʼutujil, Kakchiquel, and Spanish	2 (6%)
Literacy	
Can read and write	23 (70%)
Can’t read nor write	8 (24%)
Can read but not write	1 (3%)
Can somewhat read and write	1 (3%)
Education	
None	10 (30%)
1st grade	1 (3%)
2nd grade	3 (9%)
3rd grade	3 (9%)
5th grade	1 (3%)
6th grade	3 (9%)
9th grade	1 (3%)
High School Diploma equivalent	1 (3%)
Upper High School: no diploma	4 (12%)
University	6 (18%)

**Figure 1:**
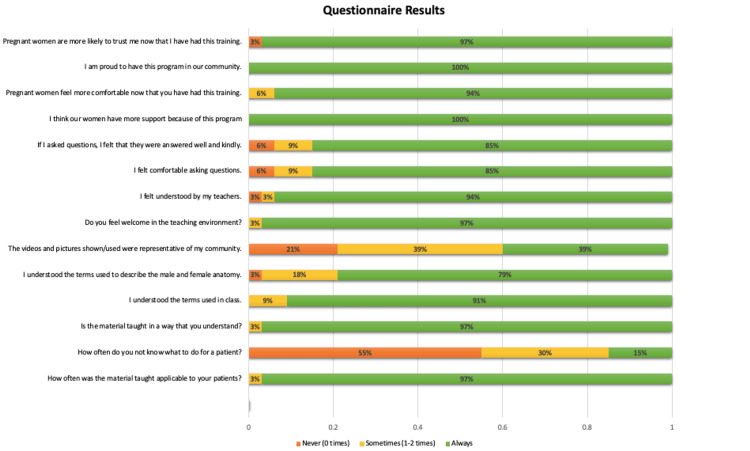
Questionnaire Results (n=33) This details the responses to the questionnaire. The relative percentages of answers are indicated on the image.

Figure 1 illustrates the results of the 14-question survey. Comadronas expressed that the curriculum was generally applicable (97%) and comprehensible (97%). However, only 79% felt comfortable with the terminology used to describe male and female anatomy. When it came to the teaching tools used during TBA training, opinions were mixed. About 39% believed the tools used were always representative of their community, 39% said they were sometimes representative, and 21% felt they were never representative.

Regarding the learning environment, 97% of comadronas reported always feeling welcome, and 94% felt understood by the instructors (Table 2). However, 15% did not always feel comfortable asking questions. In terms of community impact, 94% believed that pregnant women in their communities felt more comfortable and trusted comadronas after receiving training.

**Table 2:** Quotes From Focus Group Discussion This demonstrates the theme and sub-themes quotes from the focus group discussion.

Theme	Sub-themes	Quotes
Empowerment	Support for Comadronas. Recognition of education. Increase in confidence.	“There is greater job security.” “The school provides more support for indigenous women.” “The program is inclusive of the ancestral midwives.”
Impact on Clinical Care & Outcomes	Increased skills. Improved referral rates. Improved maternal outcomes.	“So far I have not had cases where I feel unprepared.” “I can detect the baby position, the dilation count, calculate the gestational age, or detect a twin pregnancy by Doppler.” “I have more information on women’s health.” “The topics helped us explain processes to pregnant women and remove doubt and false beliefs.”
Impact on community	Increased trust . Increased likelihood to seek care.	“Our community sees that we are trained and they come to us.” “Little by little we have earned the trust of the women. Our skills have helped us care for the woman and baby. We are able to detect elevated blood pressure or problems with fetal heart rate earlier.”

Group interview results

The focus group discussion served as a dedicated forum for comadronas to openly share their program experiences and discuss topics that may have been overlooked in the survey but were deemed crucial by them. In terms of community impact, the comadronas conveyed a sense of support and recognition, citing the program's role in bolstering their confidence and enhancing their ability to deliver improved care while upholding traditional midwifery practices. The participants also expressed increased empowerment of midwives, a subjective decrease in fetal and maternal mortality, and a subjective increase in critical case referrals to city hospitals. Importantly, the comadronas consistently reported feeling welcome and understood in the program's learning environment, reflecting the program's efforts to create an inclusive and culturally respectful atmosphere.

“I am very happy and at peace with my work now. In the past, the comadrona had no tools. She just sat there and waited for the baby. Now, if a woman comes to me for prenatal care, I can educate her and help her. Other midwives patients come to me as well. Thank you. I never had supplies in the past. I used to tie the baby cord with thread. Now I have clean gauze and clamps. I was part of the 3rd graduating class, and I thank you. Now we know the warning signs. When we identify them, we refer. If we didn't have these supplies, we would not know what is going on inside the woman. We can save the lives of the babies now.”

“We are feeling the positive impact from the school we are all trained in. All of us here at the birthing center completed the program. Our community sees that we are trained, and they come to us. All the referrals we make to the hospital are actual emergencies that have saved lives. If it weren't for the school, we would make many errors in caring for the women and referring."

Although the participants did not have any adverse impacts to report, the group interviews proved instrumental in providing constructive feedback. Recommendations included the provision of additional resources such as stethoscopes and blood pressure cuffs, the implementation of review sessions for knowledge reinforcement, more representative videos, and offering more hands-on training opportunities. Finally, the women expressed a desire for a closer relationship between comadronas and the Ministry of Health (MOH). Historically, the MOH has shown reluctance to embrace and endorse traditional practices, leading to strained relations. There has been improvement in the relationship, as shown by the MOH signing the School of PowHER graduation certificates. However, the comadronas hope that this program can foster better communication and garner support from the MOH. More information can be found in Appendix, Tables 3, 4.

## Discussion

The School of PowHER is a pioneering initiative designed to reduce maternal morbidity and mortality rates by bridging cultural divides and improving maternal health in indigenous communities. Guatemala's indigenous culture is deeply rooted in longstanding traditional practices that believe traditional healers and comadronas possess Mayan ancestral medical lore, and their legitimacy is rooted in the trust placed in them by their indigenous community [9]. This study demonstrates that while comadronas are already experts in Mayan culture, effective clinical training further elevates their role, securing recognition and legitimacy within their communities and the formal Guatemalan healthcare system. By having the Ministry of Health and Public Assistance (MSPAS) Director of the Department of Solola sign the graduation certificates, Saving Mothers and the MSPAS validate the comadronas and their work. This acknowledgment marks a crucial step toward bridging the gap between ancient cultural beliefs and the biomedicalization of healthcare.

Although concerns emerged regarding terminology of anatomy, particularly in reproductive health discussions, the School of PowHER encourages the trainees to learn the proper medical terminology as a way to help further bridge the gap between the biomedical Western world and the comadrona. Approximately one-third of the women did not feel comfortable with the terminology used to describe male and female anatomy. This discomfort likely originates from multiple socio-cultural factors, most especially conservative religious influence and lack of reproductive health literacy. Both the Catholic and Evangelical churches have long exercised their influence within local and national governments in Guatemala [11]. In relation to programs dealing with reproductive health, religious organizations pose a barrier. This is due to their conservative nature on more taboo subjects such as sexuality and family planning and can stall comprehensive sexuality education curriculum development or implementation processes, especially when such groups wield power close to or within the government [12].

Mayan languages are not recognized as co-official by national governments and are often stigmatized by the non-Maya [13]. They are generally absent from national state institutions, universities, and businesses, and their public use is limited to the local community, regional markets, traditional institutions, and/or the household [13]. The Mayan’s low socioeconomic status in Guatemala also leaves them at an educational disadvantage, possibly contributing to the lack of patient knowledge and their ability to properly communicate health concerns [14]. Until the 1990s in Guatemala, Mayan children were discouraged or punished for speaking their native language in school. Mayan languages were excluded and stigmatized by non-indigenous teachers. Intercultural bilingual education programs introduced in the 1990s aimed to align educational materials with local norms and sensitivities, making them resonate with the target audience [13].

The lack of direct translations in Mayan dialects further complicates communication and reflects cultural and religious beliefs. Isihuayo is the Nahuatl term to describe the “female part,” an organ situated in the lower torso of a woman believed to have roots radiating out from it [15]. Although Isihuayo traditionally described a specific part of the woman’s body, over the last few years, especially with the contact that the women have increasingly had with biomedicine, this term is now also equated with two other organs in the lower torso: matriz (uterus) and vejiga (bladder), both terms are in Spanish. Reproductive health education in secondary schools is conducted in Spanish, despite many children not speaking Spanish at home or having these conversations with their parents. It highlights the need to balance cultural sensitivity in educational materials while avoiding misinformation and stigma.

Despite these challenges, this study reveals that the program is culturally sensitive and effective, with comadronas rating it highly for applicability (97%) and comprehensibility (97%). The curriculum is taught in their native language by members of their own community and is open to all literacy levels. About 97% of comadronas reported always feeling welcome, and 94% felt understood by the instructors. The group interviews played a critical role in emphasizing the program's positive outcomes, encompassing heightened confidence among participants, improved patient care, and a perceived decrease in maternal and fetal mortality rate. Their belief that pregnant women in their communities felt more comfortable and trusted them more after receiving training highlights that the program was well received by the community. Their training has addressed critical gaps in prenatal care and safe labor practices.

Participants also offered constructive feedback to enhance the program, emphasizing the need for more culturally relevant teaching materials, additional resources, and stronger collaboration with the Ministry of Health. Prioritizing cultural relevance, bolstering partnerships, and incorporating participant feedback into program improvements are pivotal to the School of PowHER’s sustained impact. Surveys administered to future cohorts will support progress monitoring and inclusivity. The program’s culturally sensitive and community-centered approach can serve as a model for similar initiatives in other low- and middle-income countries grappling with high maternal mortality rates. Despite challenges such as language barriers and participant access, this study underscores the profound potential of culturally appropriate healthcare programs to advance maternal health equity on a global scale.

Limitations of our study include the significant language barriers, demographic challenges, and access to participants, leading to a low number of total included participants in this study. The surveys were first created in English. The surveys were originally crafted in English and subsequently translated into Spanish by a native speaker, but it is important to note that although all study participants spoke Spanish, this was their second language, but it is important to note that the dialects may differ.

Additionally, many of the students only spoke Tzʼutujil, requiring further assistance. The literacy levels also varied greatly, many could not read or write, and required verbal translation of the questions. Due to the geographical challenges, we could not bring all of the students to the research site. In addition, the program educators were present and the participants may have been less likely to voice their concerns. Regardless of these limitations, the cultural appropriateness of the program was successfully examined.

## Conclusions

The School of PowHER serves as a promising model for culturally sensitive healthcare education programs. Its inclusive design and adaptability across diverse cohorts demonstrate its potential to enhance healthcare outcomes in various communities. This study's findings, highlighting increased confidence, improved patient care, and perceived reductions in maternal mortality, underscore the program's effectiveness. Moreover, the feedback calling for more culturally relevant materials and stronger collaboration with the Ministry of Health suggests areas for further enhancement. By adopting a community-centered approach, this program can inspire similar initiatives in low- and middle-income countries, particularly those grappling with high maternal mortality rates, while addressing language and access challenges.

## Appendices

**Table 3:** Questionnaire Percent Responses This table indicates the questionnaire percent responses to respective questions.

	Responses (count and %)		
Questions	Never (0 times)	Sometimes (1-2 times)	Always
How often was the material taught applicable to your patients?	-	1 (3%)	32 (97%)
How often do you not know what to do for a patient?	18 (55%)	10 (30%)	5 (15%)
Is the material taught in a way that you understand?	-	1 (3%)	32 (97%)
I understood the terms used in class.	-	3 (9%)	30 (91%)
I understood the terms used to describe the male and female anatomy.	1 (3%)	6 (18%)	26 (79%)
The videos and pictures shown/used were representative of my community.	7 (21%)	13 (39%)	13 (39%)
Do you feel welcome in the teaching environment?	-	1 (3%)	32 (97%)
I felt understood by my teachers.	1 (3%)	1 (3%)	31 (94%)
I felt comfortable asking questions.	2 (6%)	3 (9%)	28 (85%)
If I asked questions, I felt that they were answered well and kindly.	2 (6%)	3 (9%)	28 (85%)
I think our women have more support because of this program	-	-	33 (100%)
Pregnant women feel more comfortable now that you have had this training.	-	2 (6%)	31 (94%)
I am proud to have this program in our community.	-	-	33 (100%)
Pregnant women are more likely to trust me now that I have had this training.	1 (3%)	-	32 (97%)

**Table 4:** Focus Group Questions This table details the open-ended questions asked during the focus group interview. PowHER: Providing Outreach in Women’s Health and Educational Resources, CAP: Centro de Atención Permanente.

Group Discussion Questions:
The instruments or tools that we teach with, do you use them in your care?
How does this program affect how you feel?
Do you feel happier now that you have completed the program?
Do you feel more confident in your care for women now that you have completed the program?
Do you feel more connected to your community now that you have completed this program?
How do you feel about the School of PowHER’s effect on your community?
Please describe your experience as a student in the School of PowHER.
The pictures we used for training, do they apply to your patients?
The pictures we used for training, do they make sense?
How do you think the School of PowHER changed your community in a positive way?
How do you think the School of PowHER changed your community in a negative way?
What would you improve about the program?
Have your referrals to the CAP or hospital increased after completing the program?
How did the School of PowHER affect your care for women?
What are your opinions on the way we teach at the School of PowHER?
What things do you feel well prepared for after your training?
What things do you feel unprepared for even after your training?
If you provided care to women before your training, how has your education changed how you care for women?
